# Gene Expression Profiles Suggest a Better Cold Acclimation of Polyploids in the Alpine Species *Ranunculus kuepferi* (Ranunculaceae)

**DOI:** 10.3390/genes12111818

**Published:** 2021-11-18

**Authors:** Eleni Syngelaki, Claudia Paetzold, Elvira Hörandl

**Affiliations:** 1 Department of Systematics, Biodiversity and Evolution of Plants (with Herbarium), Georg-August-Universität Göttingen, 37073 Göttingen, Germany; elvira.hoerandl@biologie.uni-goettingen.de; 2 Department of Botany and Molecular Evolution, Senckenberg Research Institute, 60325 Frankfurt am Main, Germany; claudia.paetzold@senckenberg.de

**Keywords:** alpine plants, cold stress, DNA methylation, gene expression, Gene Set Enrichment Analysis, geographical parthenogenesis, polyploidy, *Ranunculus kuepferi*

## Abstract

Alpine habitats are shaped by harsh abiotic conditions and cold climates. Temperature stress can affect phenotypic plasticity, reproduction, and epigenetic profiles, which may affect acclimation and adaptation. Distribution patterns suggest that polyploidy seems to be advantageous under cold conditions. Nevertheless, whether temperature stress can induce gene expression changes in different cytotypes, and how the response is initialized through gene set pathways and epigenetic control remain vague for non-model plants. The perennial alpine plant *Ranunculus kuepferi* was used to investigate the effect of cold stress on gene expression profiles. Diploid and autotetraploid individuals were exposed to cold and warm conditions in climate growth chambers and analyzed via transcriptome sequencing and qRT-PCR. Overall, cold stress changed gene expression profiles of both cytotypes and induced cold acclimation. Diploids changed more gene set pathways than tetraploids, and suppressed pathways involved in ion/cation homeostasis. Tetraploids mostly activated gene set pathways related to cell wall and plasma membrane. An epigenetic background for gene regulation in response to temperature conditions is indicated. Results suggest that perennial alpine plants can respond to temperature extremes via altered gene expression. Tetraploids are better acclimated to cold conditions, enabling them to colonize colder climatic areas in the Alps.

## 1. Introduction

Temperature stress is affecting several developmental processes in the life cycle of flowering plants [1] and is considered a key constraint to the geographical distribution of species. Aside from distributional ranges, changes in average temperature can affect the phenology, defense capacity, growth, and development of plants e.g., [2,3,4,5,6].

Cold temperature stress is defined as chilling (0–20 °C) or freezing (<0 °C) and represents a major abiotic stress, threatening growth and development e.g., [7,8]. Most notably, it induces biochemical, physiological, structural, and morphological modifications e.g., [9,10,11,12,13] such as changes in light use, ROS production, carbon assimilation, photosynthesis rate, membrane permeability, fluidity, and cell wall architecture e.g., [7,14,15,16,17,18,19].

Flowering plants evolved various adaptation strategies to survive and reproduce under adverse temperature conditions, such as plastic responses, which are observed to be positively correlated with colonization of novel habitats e.g., [20,21] and subsequent selection of fitting phenotypes over many generations [22,23,24,25,26]. In recent decades, a growing number of studies have been focused on phenotypic plasticity, one component of which is thought to be changes in gene expression patterns, and its evolutionary aspects e.g., [27,28,29,30,31,32,33]. A reliable subset of them focuses on the triggering role of environmental conditions [34,35] and the capacity of individuals for phenotypic accommodation [23,25] as well as acclimation to the new conditions [36].

The molecular response of plants towards environmental conditions is dynamic and extremely complex, as a typical plant cell possesses more than 30,000 genes [37]. Alterations on a phenotype can be depicted in morphology, physiology, and gene expression, as single changes or a combination of these [25,32,38,39,40]. The genes involved in these procedures can be induced by cold *per se* or by the relative state of dehydration following cold stress [41]. As plants are sessile organisms, the effects of cold stress seem to be of great importance regarding the acclimation to environmental conditions e.g., [39,42,43], with timing, combination, and intensity of the stress parameters presumably playing an important role e.g., [44].

Cold acclimation, i.e., the acquisition of increased freezing tolerance upon prior exposure to non-lethal low temperatures [45,46,47], is a sophisticated mechanism plants evolved to endure cold stress. It is moderated via structural and functional remodeling. Profound changes in gene expression profiles affect the composition of the transcriptome, proteome, and metabolome e.g., [7,48]. Gene expression depicts the way phenotypes are determined under particular environmental conditions [49]. The combination of environment and genotype influences the expression of a phenotype in a world of continuously changing conditions [39].

Polyploidy has several effects on vigor, physiology, morphology and other adaptive traits and can result in increased survival fitness in harsher environments [50,51,52,53]. Polyploids are specifically more abundant in high latitudes and regions with colder climates [54]. It is hypothesized that under cold temperature conditions polyploid plants are reducing cell numbers and increasing cell size [51], thus adjusting their growth and exposure of reproductive tissues towards the putative adaptive morphology of alpine dwarfism [13]. The shift to asexual reproduction modes in some polyploids and epigenetic modifications are suggested to further improve their adaptation to stress conditions [13,55,56].

Epigenetic modifications, such as DNA methylation, histone modifications, and chromatin rearrangement can directly or obliquely regulate gene expression e.g., [57,58,59,60,61]. In plants, DNA methylation is a documented epigenetic mechanism, which could mediate phenotypic plasticity within a single generation [62] and between generations [63]. DNA methylation changes can be induced by environmental stimuli, either biotic or abiotic e.g., [64,65,66], while DNA methylation profiles could be somewhat fixed e.g., for transgenerational inheritance e.g., [67,68,69,70]. Furthermore, DNA methylation changes, e.g., gene silencing, can also be triggered by genomic stresses, such as polyploidization and hybridization, which could also result in genome-wide transcriptional rewiring e.g., [50,71,72,73,74,75]. Thus, DNA methylation can be beneficial in the procedures following polyploidization events concerning the re-establishment of genomic balance and structural and functional remodeling [76,77,78,79].

Phenotypic plasticity, here defined as the ability of a single genotype to differentially respond to environmental stimuli [28,80], is thought to be under genetic and epigenetic control e.g., [43] and often correlated with transcriptional differentiation [33,81]. An epigenetic background of phenotypic plasticity suggests that DNA methylation provides a plant with a more rapid reaction to variable environmental conditions compared to DNA mutation. However, the resulting phenotype is not necessarily stable [34].

Most studies on gene expression under temperature stress were so far conducted on annual model organisms such as *Arabidopsis thaliana* or on crop plants e.g., [47,82,83]. Little is known on the plasticity of perennial plants growing under natural, extreme conditions. *Ranunculus kuepferi* Greuter & Burdet is an alpine perennial herb appropriate for studying acclimation to cold conditions. The species is primarily distributed across the European Alps, in altitudes between 1300–2800 m [84,85,86,87,88] along a pronounced geographical parthenogenesis scenario [89]. Geographical Parthenogenesis in general describes related sexual and asexual organisms with different geographical distributions [90]. In *R. kuepferi*, diploid plants are predominantly sexual and restricted to the warmer Southwestern Alps, whereas autotetraploid plants are facultative apomicts (aposporous), with varying proportions of sexual and asexual seeds, and colonize previously glaciated areas, i.e., the northern, central and eastern Alps as well as the northern Apennines and Corsica [84,85,88,91]. Tetraploid populations occur at higher elevations in the European Alps than diploids and exhibit a pronounced niche shift towards colder temperatures [87,88]. High alpine habitats are characterized by short growth periods and cold spells, eventually with nocturnal frost during flowering time (spring or summer). It was suggested that the niche differentiation between the cytotypes is associated with a combination of climatic conditions and reproduction mode, with the asexual taxa having a distributional advantage towards cooler conditions [92].

Previous studies revealed that the tetraploid cytotype originated 10–80 kyears ago [92], presumably by multiple and recurrent autopolyploidization events [86,93]. The genetic differentiation between cytotypes, regardless of reproduction mode, is very low and on a similar level within cytotypes (F_st_ values ~0.3 for both cytotypes) [89]. The epigenetic background of the species proposes a differentiation of the cytotypes, but also a correlation of abiotic environmental conditions with the epigenetic variation in natural populations and in experimental conditions [94,95]. Thus, a putative epigenetic background of the niche shift of the tetraploids in the Alps is indicated, with epigenetic variation being associated with elevation in natural populations and higher persistence under cold treatments [94,95]. Syngelaki et al. [96] highlighted the potential of phenotypic plasticity, with changes of growth parameters linked to DNA methylation patterns, for acclimation to environmental conditions. These experiments confirmed the different niche preferences of cytotypes in natural populations, as diploids grow better under warm conditions, while tetraploids perform better in cold treatments.

Herein, we employed diploid and tetraploid plants already used by Syngelaki et al. [95,96] and exposed them to different controlled temperature treatments, to assess the gene expression profiles of individuals. We aim to investigate whether cold temperature stress influences gene expression and try to gain further insights into the expression dynamics. A temperature-sensitivity of gene expression profiles is speculated, which could act as a rapid response towards stressful environments. We also investigated the differentiation of the gene expression profiles according to the ploidy level of the individuals. We hypothesize that the observed niche shift of the tetraploid cytotype has the physiological background of a better cold acclimation. Finally, we associated the gene expression results with DNA methylation, on a transcriptomic level, as a correlation between them would explain the potential of tetraploid *R. kuepferi* to adapt to cold conditions at higher altitudes during the postglacial recolonization of the European Alps.

## 2. Materials and Methods

### 2.1. Plant Material and Experimental Design

Diploid and tetraploid plants of *R. kuepferi* used in the present study were part of a long-term temperature stress experiment, as described in Klatt et al. [97] and Syngelaki et al. [95,96]. Plants were collected throughout the distribution range of the species in the European Alps [87] during the growing seasons of 2013 & 2014. Subsequently, the plants were re-potted in garden soil at the Old Botanical Garden of Göttingen University, where their ploidy level was defined via Flow Cytometry of silica gel dried leaf material collected in the field [88]. For the scope of the current experimental design, which was conducted from 2014 onwards, the plants were exposed to different temperature conditions during the sprouting and flowering period, while the rest of the parameters were kept equal. The settings for the transcriptome study were as in Syngelaki et al. [95,96] (Table 1). The temperature conditions were simulating the natural environment of the species in the Alps, including freezing during some nights.

Altogether 262 individuals were categorized into four groups corresponding to their ploidy level and treatment: cold diploids (CD, 63 plants), warm diploids (WD, 79 plants), cold tetraploids (CT, 71 plants), and warm tetraploids (WT, 49 plants). At the peak of the flowering season in beginning of summer 2019, leaf tissue was collected simultaneously from three individuals per group, each originating from different natural populations, immediately frozen in liquid nitrogen and stored in −80 °C. Sample collection localities can be found in Supplementary Table S1.

### 2.2. RNA Extraction and Sequencing

Frozen leaf tissue was pulverized in liquid nitrogen and a maximum of 100 mg of powder was used for RNA isolation with the RNAeasy Plant^®^ Mini Kit (QIAGEN, Hilden, Germany) following the provided protocol. RNA quantity and quality were determined with Nanodrop, a Qubit^TM^ with the RNA HS Assay Kit (ThermoFisher Scientific, Waltham, MA, USA) and an Agilent 2100 Bioanalyzer (Agilent Technologies, Santa Clara, CA, USA). Library preparation and sequencing with HiSeq 4000 (Illumina, San Diego, CA, USA) was conducted at the Transcriptome and Genome Analysis Laboratory of the Microarray & Deep-Sequencing Core Facility (UMG, Georg-August-Universität, Göttingen, Germany) producing 50 bp single end reads.

### 2.3. Bioinformatics

The quality of reads was assessed using FastQC v.0.11.4 [98]. Raw reads were trimmed with CutAdapt v.2.3 [99], removing adapters and bases with a phred score below 30 and removing reads shorter than 30bp after trimming. As there is no available genomic reference for *R. kuepferi*, the transcriptomes from all diploid individuals were pooled for a de novo assembly with Trinity v.2.8.6 and default parameters, except for max. memory was set to 50Gb [100,101] to produce a pseudoreference. The quality of the resulting assembly was verified with BUSCO v.3.0.2 [102] (Supplementary Figure S1). TransDecoder v.5.5.0 [103] was used to identify the longest Open Reading Frame per assembled contig. Coding sequences were annotated using the blastp algorithm under NCBI-BLAST v.2.10.0 [104] and the December 2020 release of Uniprot as reference. Annotation reports were produced using Trinotate v.3.2.1 [105]. Trimmed reads of each sample were mapped against the annotated pseudoreference individually using Bowtie2 v.2.3.5.1 with default parameters [106].

Raw counts of mapped reads were calculated employing the Rsubread/Subread package v.2.4.0 [107] in R/Bioconductor (v.4.0.3 and v.4.1.0/v.3.12 and v.3.13, respectively) [108] in R Studio [109]. Resulting matrices were further analyzed with DESeq2 v.1.30.0 [110] with a false discovery rate (FDR) threshold of < 0.05 and the Benjamini–Hochberg p-value normalization adjustment [111]. Loci were identified as differentially expressed regarding the comparison of interest (cytotypes, treatments), with the group of warm diploids (WD) used as reference, and were visualized in a heatmap with ggplot2 v.3.3.5 [112].

ClusterProfiler v.4.0.2 [113] was used for Gene Set Enrichment Analysis. This package currently only accepts single organism references via AnnotationDbi v.1.55.1 [114]. Of the currently accepted reference genomes, *A. thaliana* is most closely related to *R. kuepferi* [115]. Consequently, the pseudoreference was annotated again as described above using the updated TAIR 10 release [The Arabidopsis Information Resource (TAIR), http://www.arabidopsis.org; accessed on 11 March 2021] and the resulting annotations were employed in ClusterProfiler for a separate run for each cytotype, checking all subontologies and with several 1 Mio. permutations. For these analyses, the warm treatment was set as the control condition. Dot plots were generated with enrichplot v.1.13.1 [116].

### 2.4. qRT-PCR

Quantitative real-time RT-PCR was conducted to validate the differential gene expression revealed by bioinformatic analyses. The annotation reports of the pseudoreference were screened for possible genes of interest (GOIs), which are stated to be related, directly or indirectly, to DNA methylation and gene regulation [12,60,61,82,117,118,119,120,121,122,123], as well as housekeeping genes [124,125]. Primers were designed for two methyltransferases (CAMT3, PMT2), two demethylases (JM706, JMJ25), the AGO4B gene, which is participating in the RNA-directed DNA methylation (RdDM) pathway in *Arabidopsis* and rice [121] and the housekeeping gene *Actin* (Supplementary Table S2). Primer specificity was tested with a touch-down PCR; products were sequenced and compared to the respective gene in the pseudoreference.

Complementary DNA synthesis and qRT-PCR was performed using QuantiTect Reverse Transcription Kit (QIAGEN, Hilden, Germany) and the Rotor-Gene SYBR Green PCR kit (QIAGEN, Hilden, Germany) in QIAGEN Rotor-Gene Q cycler, equipped with Q-Rex Software and following the instructions of the manufacturer for two cycling steps and a 45 cycles PCR program for three technical replicates per sample. To evaluate the differential gene expression, the amplification performance as the log of fold change was calculated with the ΔΔCt method [126] in Excel 2016, using the housekeeping gene *Actin* as endogenous control for normalization and warm diploid individuals were considered the reference.

## 3. Results

### 3.1. Pseudoreference and Mapping

In the current study, gene expression profiles of six diploid and six tetraploid individuals of *R. kuepferi,* under cold (stress) and warm (control) temperature treatments were explored. Sequencing of the samples resulted in a mean of 31,918,319 raw reads per sample with a mean of 31,707,039 reads retained after trimming. The assembly of the pseudoreference resulted in 71,444 transcripts, with 15,224 of them functionally annotated. Through Bowtie2 mapping, we obtained an average mapping rate of 93.08% per sample (Table 2).

### 3.2. Differential Gene Expression

A total of 2617 significantly differentially expressed genes were identified between the four predefined groups. Among all groups, more genes were found to be down-regulated compared to up-regulated (Table 3).

**Figure 1:**
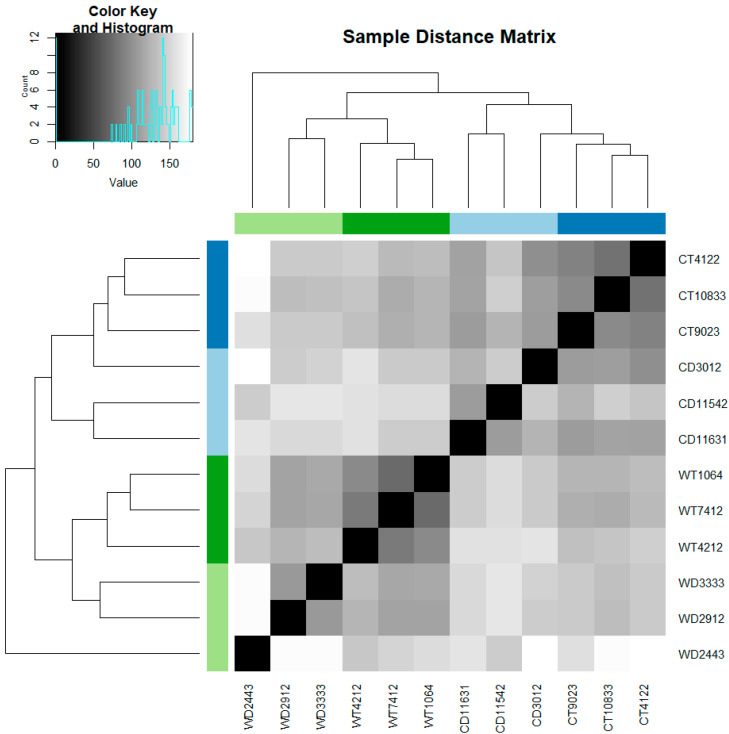
Heat map showing the expression of transcriptomes of diploid and tetraploid *R. kuepferi* plants under warm (control) and cold (stress) conditions. Regarding the sample IDs the capital letters stand for the predefined groups (WD: warm diploid, CD: cold diploid, WT: warm tetraploid, CT: cold tetraploid) and the numbers stand for the populations in the wild (Supplementary Table S1).

Gene expression was correlated with temperature, while the ploidy level of the plants under the same environmental conditions did not affect the gene expression strongly (Figure 1). One WD individual (WD2443) seems to present gene expression patterns different from all the other samples. The same individual seems to be an outlier also in the qRT-PCR analysis.

### 3.3. Gene Set Enrichment Analyses

**Figure 2:**
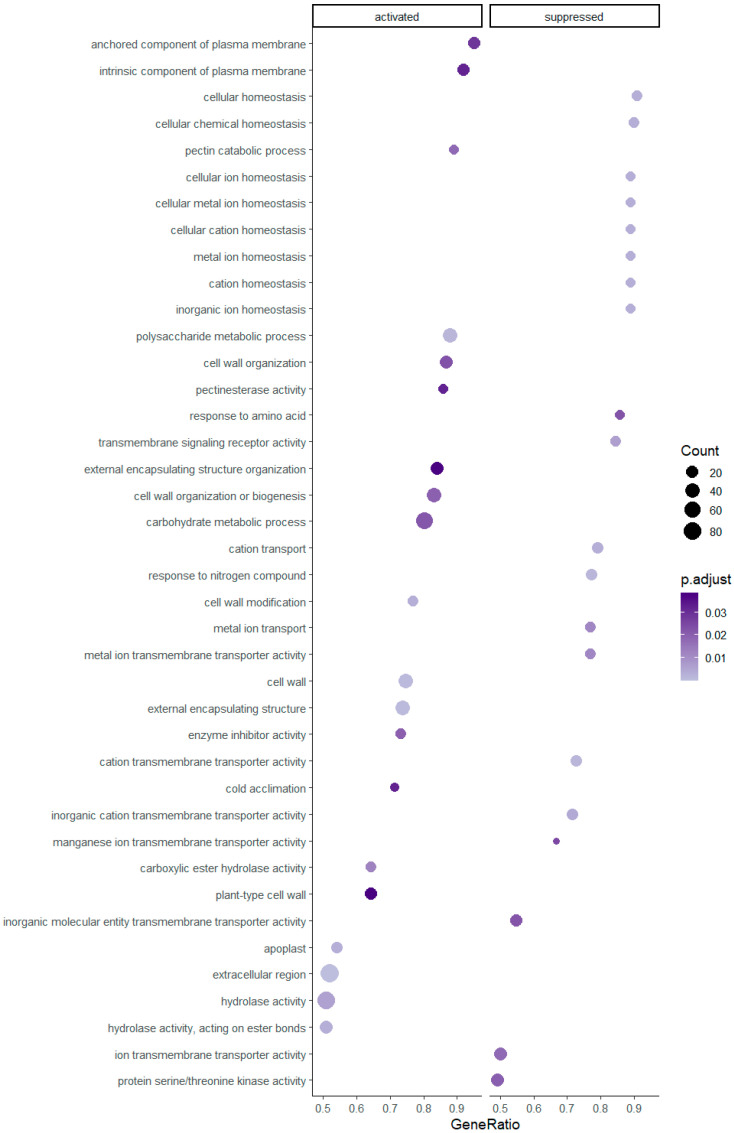
Significantly enriched gene set pathways resulting from the analysis of differentially expressed genes in diploid individuals of *R. kuepferi*.

**Figure 3:**
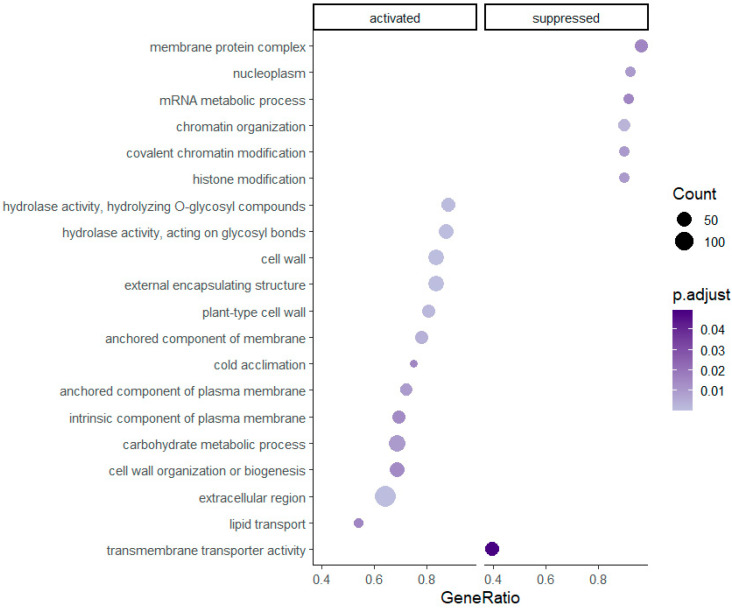
Significantly enriched gene set pathways resulting from the analysis of differentially expressed genes in tetraploid individuals of *R. kuepferi*.

Gene set enrichment was successfully assigned and statistically significant for 59 pathways in the diploids and 20 pathways in the tetraploids. Enriched gene sets with higher GeneRatios, which in ClusterProfiler are defined as ‘count/setSize,’ where ‘count’ is the number of genes that belong to a given gene set, while ‘setSize’ is the total number of genes in the gene set, are presented in Figure 2 and Figure 3. Regarding Figure 2, an extract of all resulted enriched pathways is shown due to graphical purposes and a dotplot of all pathways is provided in Supplementary Data (Supplementary Figure S2). Overall, 25 and 13 pathways have been activated in diploid and tetraploid individuals, respectively. Pathways which are linked to the plasma membrane e.g., ‘anchored component of plasma membrane’, ‘(cation) transmembrane transporter activity’ and the cell wall e.g., ‘cell wall organization or biogenesis’, ‘plant-type cell wall’, as well as the ‘cold acclimation’ and ‘hydrolase activity’ pathways, are activated in both cytotypes (Figure 2 and Figure 3). However, pathways related to ion/cation homeostasis and enzymic activity, such as ‘(cellular) metal ion homeostasis’ and ‘protein serine/threonine kinase activity’ are enriched only in diploids, while pathways such ‘histone/chromatin modification’ and ‘lipid transport’ are enriched only in tetraploid individuals.

### 3.4. Genes of Interest and qRT-PCR

A total of 38 genes of interest, identified by their involvement in DNA methylation, were significantly differentially expressed among the four predefined groups (Table 4).

The correlation of differential gene expression analysis with epigenetics was validated via qRT-PCR. For almost all individuals the expression of all five of the selected genes was down-regulated (Supplementary Table S3), corresponding to the differential expression analysis results of DESeq2. Only for one individual, an up-regulation of all the genes was detected. This individual is the same that separates from the rest of the samples under cold treatment (Figure 1).

## 4. Discussion

Plant acclimation to cold stress induces various cellular processes through a cascade of change in gene expression and protein synthesis e.g., [127,128]. It is estimated that between 4% and 12% of the transcriptome of *A. thaliana* changes after a respective period of several hours, days or weeks of exposure to chilling temperatures [129,130]. This differentiation in gene expression combined with the observation of different gene clusters being up-regulated during different times of the stress exposure indicates a hierarchy in the functional response, with signaling of harmful conditions or increasing freezing tolerance comes first [129,131,132], while circadian clocks being hypothesized to play an important role in general regulation [117,133]. Polyploidy may affect cold acclimation as polyploids are thought to perform better under cold conditions e.g., [54]. We analyzed here for the first time gene expression of a perennial alpine plant under different temperature conditions, and evaluated effects of different ploidy levels on the response to cold stress.

### 4.1. Ploidy Effects on Gene Expression and the Distribution Pattern

The ploidy level of the individuals *per se* does not appear to be a significant contributor to the observed differential gene expression (Figure 1). This differs from previous studies on DNA methylation patterns, mode of reproduction, and morphological traits of *R. kuepferi* which revealed significant ploidy effects [95,96,97]. The congeneric species of the *Ranunculus auricomus* complex also showed strong ploidy effects in gene expression profiles of ovules [134]. In our study, the main effects in gene expression changes are due to treatments, not to ploidy. In autopolyploids, transcription profiles can be influenced by a multitude of factors, which are caused by genome duplication e.g., dosage effects due to the presence of additional copies of genes [78]. However, autotetraploid rice does seemingly not show a genome-wide dosage effect on gene expression; likely because subfunctionalization maintains the functional pleonasm of duplicated genes and avoids any energy waste [135,136,137,138]. Regarding the WD individual, which is an outlier for both the gene expression profiles and qRT-PCR analysis, there was no indication of lower RNA quality during wet and dry lab manipulation of the specimen. Probably, the conditional plasticity and the gene-environment interaction [39,139] of the individual, as well as the micro site of origin of the natural population, which belongs to the sympatric zone of the two cytotypes [88], could play the major role for its observed phenotype.

To investigate further how the two cytotypes cope with stress conditions, gene set enrichment for each ploidy level was assessed. Several key regulatory pathways and their interactions have been documented previously e.g., [37,117,140,141,142]. In *R. kuepferi*, diploids respond more intensively to temperature treatments compared to the tetraploids, as a higher number of gene sets is significantly differentially expressed (Figure 2 and Figure 3). Hence, diploids appear to be more stressed by cold conditions than tetraploids. The cold stress treatments of our experiments are quite similar to the natural habitat conditions of the tetraploid *R. kuepferi* plants in the Alps [95]. As the cold adapted genotypes are thought to have a distinct advantage over non-adapted ones in frost-prone environments, such as high mountain areas [9], we hypothesize that the postglacial colonization of the Alps by autotetraploid populations of *R. kuepferi* [88] did rely to a large extent on the phenotypic variation towards the climatic conditions, pronounced also as differential gene expression [143]. Thus, the geographical parthenogenesis scenario, which interrelates the cytotypes with different ecological backgrounds [88,92], is further supported. Results support the simulation study of postglacial recolonization in the Alps, which identified the acclimation/adaptation of tetraploids to a colder climatic niche in higher and more northern parts of the Alps as one decisive character of the geographical parthenogenesis pattern [92]. Our results here suggest that this niche shift has a direct physiological background of cold tolerance and is less likely due other characteristics of alpine habitats (like a lower pathogen pressure, among others).

### 4.2. Functional Aspects of Gene Expression Related to Cold Acclimation

Although some of the differentially expressed gene sets overlap between ploidy levels, there are some characteristics for either group. In diploids, most pathways are linked to ion/cation homeostasis and activity and are suppressed. Additional pathways are connected to the plasma membrane, cell wall, and hydrolase activity. Similar pathways are present in tetraploid individuals, with a greater focus on membranes and cell wall. In contrast to diploids, the tetraploids suppressed only six pathways, mostly related to chromatin and histone modification. In both cytotypes the cold acclimation pathway is activated.

As access point of the cell, membranes are injured by adverse environmental conditions, yet their stability contributes to cell behavior and activity maintenance [16,117,144]. Pathways related to cell lipid composition, such as the ones activated in the tetraploids, play an important role in the maintenance of plasma membrane functionality e.g., [145,146] and are affecting the downstream expression of genes linked to resilience to lower temperature [147]. Maintenance of plasma membrane functionality is especially important for freezing tolerance in alpine plants, as a fluid membrane allows transfer of water from the protoplast into the intercellular space, where extracellular ice nucleation takes place, leaving the protoplast unfrozen [13]. We suppose that our short freezing treatments (−1 °C during three nights per week) induced these expression changes. However, we observed no apparent damage of leaves in cold treatments [96], and hence both cytotypes are tolerant to short-term freezing and thawing during the day.

Decreasing membrane fluidity, coupled with its interaction with the cell wall, is considered to be one of the first cold sensors [148,149,150]. The connection of membrane rigidification to cytoskeletal rearrangements, calcium influxes and kinases, acts as trigger for the subsequent low-temperature response e.g., [151,152]. Changes in the composition of the cell wall can strongly affect plant stress resistance [153] as stress can up-regulate the expression of e.g., expansins and xyloglycan-modifying enzymes, which are acting on cell wall remodeling [154]. The hydrolase activity pathways activated in both ploidy levels in *R. kuepferi* under cold stress may indicate their unique roles in cell wall modification [155,156]. The cytoskeleton is also affected by cold stress [150,157] and its interactions with membranes and the cell wall play a distinctive role in cold stress tolerance [18,47,158]. The ‘cell wall organization or biogenesis’ pathway, which is activated in both diploid and tetraploid *R. kuepferi* individuals (Figure 2 and Figure 3) is relevant to the cytoskeleton and its modifications under cold stress.

The pathways of ion/cation homeostasis and activity, as well as the serine/threonine protein kinase activity are thought to be temporal and spatial events downstream of Ca^2+^ signaling e.g., [159,160]. A change in intracellular calcium ion concentration is one of the earliest signaling events triggering the response of plants to cold stress [130,161,162] with Ca^2+^ dynamics being detected within 40s after cold stress exposure [163]. Presumably Ca^2+^ channels are primary sensors for cooling rate and Ca^2+^ efflux transporters are absolute temperature sensors [164,165], while oscillations of Ca^2+^ are linked to stomatal closure in *Arabidopsis thaliana*, as a response to cold stress [166]. Furthermore, the serine/threonine protein kinases are plasma membrane receptor-like kinases (RLKs), several of which are calcium-moderated and promote the expression of cold-responsive genes (COR) through the activation of a mitogen-activated protein kinase (MAPK) cascade [153,167,168,169].

The intriguing suppression of the latter pathways, especially in diploid *R. kuepferi*, corroborates the hypotheses of tangled information encoded through Ca^2+^ kinetics, which are constantly changing as a complex mechanism of stress response and are also influenced by ‘cold memory’, i.e., former exposure to cold stress conditions [130,170]. We hypothesize that suppressed pathways of ion/cation homeostasis in diploids are probably linked to stomatal closure and consequently reduction of CO_2_ uptake/carbon gain [171]. This would explain the lower growth performance of diploids under cold conditions, as observed in the experiments of Syngelaki et al. [96].

### 4.3. Gene Expression Related to the Epigenetic Mechanism of DNA Methylation

Deciphering the epigenetic background of plants, which are exposed to abiotic stress, is a constantly developing field e.g., [43]. DNA methylation is correlated with histone proteins and their post-transcriptional modifications, as the conversion of these modifications to DNA methylation profiles is often thought to be more stable e.g., [75,172]. These interactions are associated with gene expression profiles and gene transcription in general in response to cold stress [173,174] and could encompass changes in chromatin structure and accessibility [175,176]. Chromatin remodeling has a putative function as a plant thermometer, representing a relatively direct connection between cold and gene expression [177,178]. In the present study, the pathways of histone and chromatin modifications are suppressed in the tetraploid individuals (Figure 3). Additionally, several genes, directly or indirectly related to DNA methylation, are significantly differentially expressed in both ploidy levels (Table 4). This result corroborates previous results of methylation-sensitive AFLP screenings that the cytotypes exhibit different methylation profiles [94,95].

The differential expression of genes correlated with DNA methylation is further validated by the qRT-PCR results, where the two methyltransferases (CAMT3, PMT2), two demethylases (JM706, JMJ25) and the argonaute protein AGO4B were all found to be down-regulated, as expected from the bioinformatical analyses of the transcriptomes. This fits the overall pattern of loss of methylated MS-AFLP fragments after dramatic temperature changes [95]. Methylation patterns may conserve the transgenerational epigenetic memory of response to cold treatments and hence differential acclimation and adaptation of cytotypes [94,95].

To summarize, the present study demonstrates the responses of diploid and tetraploid *R. kuepferi* plants towards cold stress in their gene expression patterns. Although both ploidy levels activate genes related to cold acclimation, the gene set pathways differ between cytotypes, suggesting a better cold acclimation of tetraploids than diploids. Consequently, our results strongly support the hypothesis of a physiological background of the observed ecological and geographical differentiation patterns between cytotypes. Altogether, cold stress induces differentially expressed gene profiles and several gene set pathways are involved in the response, either being activated or suppressed. Seemingly, these parallel mechanisms invoke energy conservation and help individuals to survive in novel and/or extreme environments. Lastly, DNA methylation is indicated to contribute to the regulation of gene expression and may preserve a different epigenetic memory for the two cytotypes.

## Figures and Tables

**Table 1:** Temperature treatment conditions during plant growth and sampling collection. Light regime was measured with a Quantum light meter (Spectrum Technologies Inc., Aurora, IL, USA) during the full light period (100% intensity) at the level of early leaf tips and first buds. Plants were rotated weekly in the cabinet to avoid effects of light and temperature gradients.

No. Plants	Cold Treatment		Warm Treatment	
	164		189	
	Diploid	Tetraploid	Diploid	Tetraploid
	74	90	92	97
Light regime (μmol m^−2^ s^−1^, SAR)	~700			
Photoperiod (h)	16; 10 of full light and 3 + 3 of twilight			
Temperature during the light/dark period (°C)				
Daytime	7		15	
Night	2 −1 (cold shocks for three nights per week)		10	

**Table 2:** Summary of sequencing and mapping data for the *R. kuepferi* transcriptomes, against the pseudoreference resulting from Trinity de novo assembly of all reads from diploid samples.

Group ^†^	Sample ID	Untrimmed Reads	Total Trimmed Reads	Mapped Reads	Mapped Reads (%)
CD	30_1_2	1,335,657	1,326,485	1,214,557	91.56
CD	115_4_2	39,260,122	39,044,345	37,240,848	95.38
CD	116_3_1	46,805,434	46,545,107	43,938,918	94.4
CT	41_2_2	37,813,775	37,625,107	34,877,792	92.7
CT	90_2_3	35,989,875	35,824,317	33,353,051	93.1
CT	108_3_3	36,888,834	36,687,214	33,980,212	92.62
WD	24_4_3	29,197,541	29,058,835	27,467,724	94.52
WD	29_1_2	34,122,764	33,708,095	31,629,139	93.83
WD	33_3_3	29,530,425	29,378,666	27,521,885	93.68
WT	42_1_2	29,277,225	29,108,345	26,919,488	92.48
WT	74_1_2	31,858,591	31,626,671	28,839,855	91.19
WT	106_4_1	30,939,580	30,551,281	27,988,255	91.61

^†^ WD: warm diploid, CD: cold diploid, WT: warm tetraploid, CT: cold tetraploid.

**Table 3:** Differentially expressed genes of *R. kuepferi* transcriptomes regarding the comparison of interest (cytotypes, treatments).

	Gene Count	Percentage (%)
Expressed genes	19,033	
Differentially expressed genes	2617	13.75
Up-regulated	1055	5.5
Down-regulated	1562	8.2
Outliers	469	2.5

**Table 4:** List of differentially expressed genes related to DNA methylation in *R. kuepferi* leaves. Annotation is according to UniProt. The genes which are highlighted are employed as genes of interest (GOIs) for the real-time qRT-PCR.

GeneID	Organism ^‡^	Function	Regulation
AGL15	BRANA	Agamous-like MADS-box protein	Down
AGL62	ARATH	Agamous-like MADS-box protein	Down
AGO1A	ORYSJ	Protein argonaute 1A	Down
AGO4B	ORYSJ	Protein argonaute 4B	Down
ALKB2	ARATH	DNA oxidative demethylase	Down
AP1	VITVI	Agamous-like MADS-box protein	Down
ATX4	ARATH	Histone-lysine N-methyltransferase	Up
CAMT3	PETHY	Caffeoyl-CoA O-methyltransferase 3	Down
CMTA3	ARATH	Calmodulin-binding transcription activator	Down
COMT1	POPKI	Caffeic acid 3-O-methyltransferase	Down
DRM1L	ARATH	DNA (cytosine-5)-methyltransferase	Down
EPFL2	ARATH	EPIDERMAL PATTERNING FACTOR-like protein 2	Down
EPFL6	ARATH	EPIDERMAL PATTERNING FACTOR-like protein 6	Down
EPFL9	ARATH	EPIDERMAL PATTERNING FACTOR-like protein 9	Down
FDM1	ARATH	Factor of DNA methylation 1	Up
JM706	ORYSJ	Lysine-specific demethylase	Down
JMJ25	ARATH	Lysine-specific demethylase	Down
LAMT	CATRO	Loganic acid O-methyltransferase	Down
MADS1	VITVI	Agamous-like MADS-box protein	Up
MBD2	ARATH	Methyl-CpG-binding domain-containing protein 2	Up
MBD6	ARATH	Methyl-CpG-binding domain-containing protein 6	Down
METE	CATRO	5-methyltetrahydropteroyltriglutamate--homocysteine methyltransferase	Down
METE2	ORYSJ	5-methyltetrahydropteroyltriglutamate--homocysteine methyltransferase 2	Down
PEAM1	ARATH	Phosphoethanolamine N-methyltransferase 1	Down
PMT1	ARATH	Probable methyltransferase PMT1	Down
PMT2	ARATH	Probable methyltransferase	Down
PMT4	ARATH	Probable methyltransferase	Down
PMT7	ARATH	Probable methyltransferase	Down
PMT8	ARATH	Probable methyltransferase	Down
PMTB	ARATH	Probable methyltransferase	Down
PMTD	ARATH	Probable pectin methyltransferase	Down
PMTI	ARATH	Probable methyltransferase	Down
PMTQ	ARATH	Probable methyltransferase	Down
PMTT	ARATH	Probable pectin methyltransferase	Down
RP6L1	ARATH	Protein RRP6-like 1	Up
RP6L2	ARATH	Protein RRP6-like 2	Up
RRP8	ARATH	Ribosomal RNA-processing protein 8	Up
SUVR1	ARATH	Probable inactive histone-lysine N-methyltransferase	Down

^‡^ Abbreviations: ARATH = Arabidopsis thaliana, BRANA = Brassica napus, CATRO = Catharanthus roseus, PETHY = Petunia hybrida, POPKI = Populus kitakamiensis, ORYSJ = Oryza sativa ssp. japonica, VITVI = Vitis vinifera.

## Data Availability

The raw sequences files are stored and available for download in the NCBI Sequence Read Archive (SRA) under BioProject PRJNA756988.

## Supplementary Materials

The following are available online at https://www.mdpi.com/article/10.3390/genes12111818/s1, Table S1. List of individuals used for the laboratory work. Listed are the sample and group ID, ploidy level, treatment, as well as country, province and altitude of origin [88,97]; Table S2. List of primers used for qRT-PCR validation; Table S3. Results of qRT-PCR and Delta-delta analyses; Figure S1. BUSCO (Benchmarking Universal Single-Copy Orthologs) plot of the *R. kuepferi* transcriptome pseudoreference; Figure S2. All gene set pathways significantly enriched from the analysis of differentially expressed genes in diploid individuals of *R. kuepferi*.
