# Supplementary material for: Gene Expression Profiles Suggest a Better Cold Acclimation of Polyploids in the Alpine Species Ranunculus kuepferi (Ranunculaceae)
Source: Genes (Basel). 2021 Nov 18;12(11):1818. doi: 10.3390/genes12111818 (PMC8625111; doi:10.3390/genes12111818)
Supplement: Supplementary file 1 [file genes-12-01818-s001.zip › genes-1457941-supplementary.pdf]

Article

Gene expression profiles suggest a better cold acclimation of polyploids in the alpine species *Ranunculus kuepferi* (Ranunculaceae)

Eleni Syngelaki, Claudia Paetzold and Elvira Hörandl

Supplementary Materials:

The following supplementary material is available for this article:

Table S1. List of individuals used for the laboratory work. Listed are the sample and group IDs, ploidy level, treatment, as well as country, province and altitude of origin [88, 97].

Sample ID	Ploidy	Temperature Treatment	Group	Country	Region	Altitude (m)
30_1_2	diploid	cold	CD	Italy	Piemonte	1743
115_4_2	diploid	cold	CD	France	Provence-Alpes-Côte d'Azur	1891
116_3_1	diploid	cold	CD	France	Provence-Alpes-Côte d'Azur	1953
41_2_2	tetraploid	cold	CT	Italy	Val d'Aosta	2174
90_2_3	tetraploid	cold	CT	Switzerland	Valais	2477
108_3_3	tetraploid	cold	CT	Switzerland	Graubunden	2171
24_4_3	diploid	warm	WD	France	Provence-Alpes-Côte d'Azur	1925
29_1_2	diploid	warm	WD	Italy	Piemonte	2020
33_3_3	diploid	warm	WD	Italy	Piemonte	2328
42_1_2	tetraploid	warm	WT	Switzerland	Valais	1789
74_1_2	tetraploid	warm	WT	Austria	Osttirol	2117
106_4_1	tetraploid	warm	WT	Italy	Trentino Alto Adige/ Südtirol	2142

† WD: warm diploid, CD: cold diploid, WT: warm tetraploid, CT: cold tetraploid

Table S2. List of primers used for qRT-PCR validation.

Gene ID	Sequence	
	Primer 5´- 3´	Primer 3´-5´
ACT	TCTCCTTGCTCATTCTGTCG	TTCCCTTTACGCCAGTGGTC
AGO4B	GTTGCTCTGAAACTAGAATG	CCACCACTTCCCTTCAAC
CAMT3	CTTTCACGATTCAACACTCC	GAACTCAGAGAAATAACAGC
JM706	AGTATGTTTGCATGGCATGTG	TCGGAGTTTGATGGGTAGTG
JMJ25	TTCATCACTGCGCTTCCATTCC	CTTGATGTGGGCACCCTGC
PMT2	GCAGTGATTGGTGTTCTCGG	GTCAGACTCATCCCTACCAGG

Table S3. Results of qRT-PCR and Delta-delta analyses.

		Hous-keeping gene	Genes of interest(GOIs)																
		ACTb	AGO4B			CAMT3			JM706			JMJ25			PMT2		
Sam-ple name	Bio-logical groups	Average Cp	Av-erage Cp	2^-(ΔΔ Ct)	log[2^-(ΔΔCt)]	Av-erage Cp	2^-(ΔΔ Ct)	log[2^-(ΔΔCt)]	Av-erage Cp	2^-(ΔΔ Ct)	log[2^-(ΔΔCt)]	Av-erage Cp	2^-(ΔΔ Ct)	log[2^-(ΔΔCt)]	Av-erage Cp	2^-(ΔΔ Ct)	log[2^-(ΔΔCt)]
30_1_2	CD	26.8	21.52	0.63	-0.2	35.47	0	-7.73	31.83	0.13	-0.9	29.1	0.23	-0.63	19.39	1.71	0.23
115_4_2	CD	29.1	19.96	9.13	0.96	38.67	0	-8	32.83	0.31	-0.51	29.52	0.85	-0.07	21.25	2.32	0.37
116_3_1	CD	26.76	21.72	0.53	-0.28	34.88	0	-7.56	32.2	0.09	-1.02	31.77	0.04	-1.45	20.65	0.69	-0.16
41_2_2	CT	26.75	22.54	0.3	-0.52	33.63	0	-7.19	35.29	0.01	-1.95	31.13	0.05	-1.26	20.29	0.88	-0.06
90_2_3	CT	27.43	22.77	0.41	-0.39	35.04	0	-7.41	31.73	0.21	-0.68	30.3	0.16	-0.81	20.78	1.01	0
108_3_3	CT	25.63	22.93	0.11	-0.98	34.94	0	-7.92	32.33	0.04	-1.4	30.89	0.03	-1.52	20.3	0.41	-0.39
24_4_3	WD	34.18	23.17	33.16	1.52	0	148764	5.17	33.6	6.18	0.79	31.31	8.37	0.92	26.01	2.9	0.46
29_1_2	WD	30.96	26.57	0.34	-0.47	0	15920	4.2	32.98	1.02	0.01	31.31	0.89	-0.05	24.92	0.66	-0.18
33_3_3	WD	29.47	27	0.09	-1.05	43.61	0	-9.37	34.18	0.16	-0.8	32.56	0.13	-0.87	23.76	0.52	-0.28
42_1_2	WT	34.14	27.65	1.44	0.16	33.26	0	-4.85	32.99	9.21	0.96	32.39	3.85	0.59	28.71	0.43	-0.36
74_1_2	WT	29.89	25.36	0.37	-0.43	37.66	0	-7.46	34.21	0.21	-0.68	32.54	0.18	-0.74	23.3	0.97	-0.01
106_4_1	WT	29.03	23.41	0.79	-0.1	37.96	0	-7.8	30.94	1.1	0.04	31.11	0.27	-0.57	22.53	0.91	-0.04
NTC	--	0	29.51	0	-10.68	0	0	-5.12	30.76	0	-8.64	31.14	0	-9.32	39.08	0	-13.76

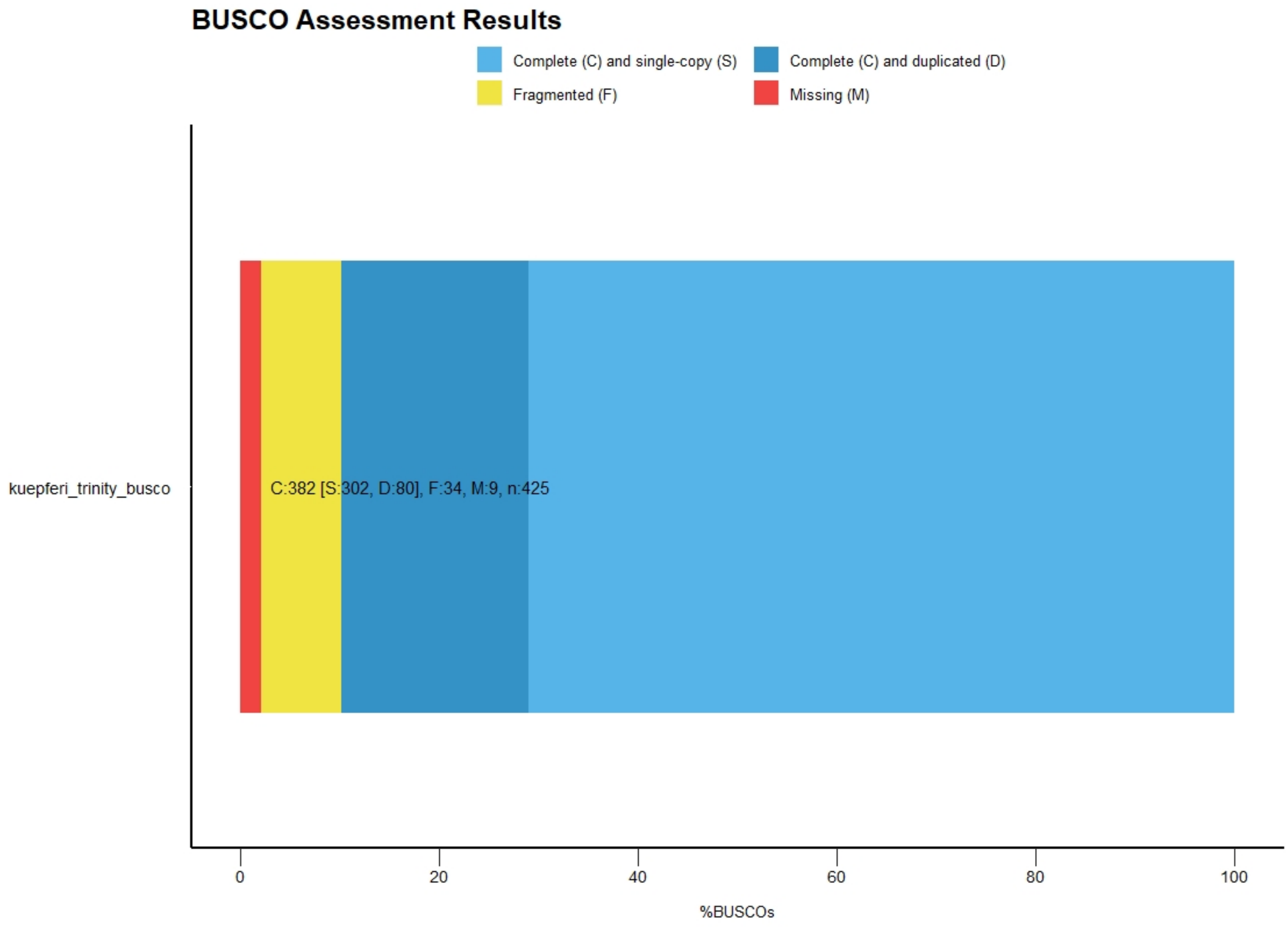

Figure S1. BUSCO (Benchmarking Universal Single-Copy Orthologs) plot of the *R. kuepferi* transcriptome pseudoreference.

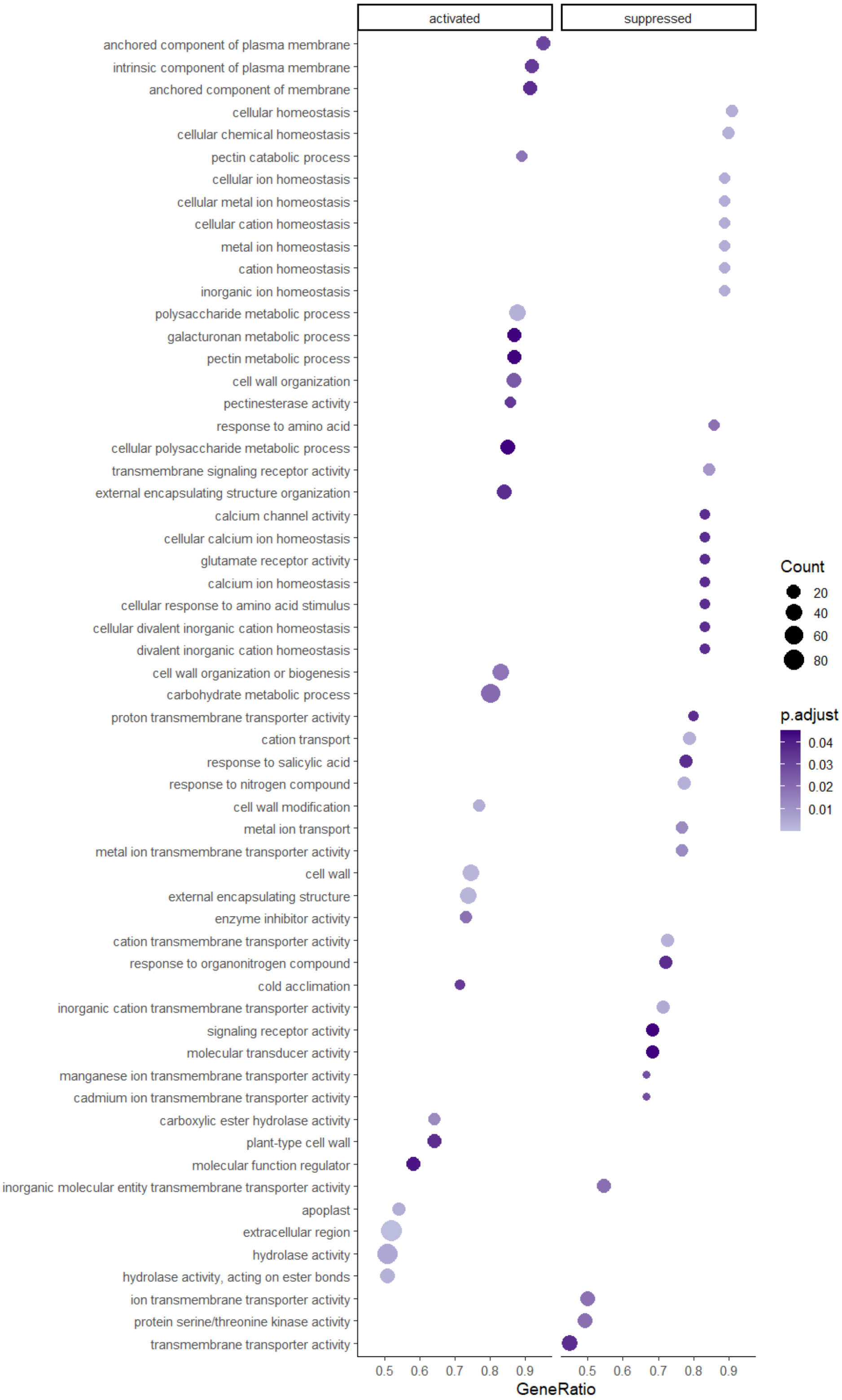

Figure S2. All Gene set pathways significantly enriched from the analysis of differentially expressed genes in diploid individuals of *R. kuepferi*.
